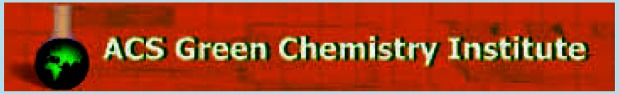# EHPnet: Green Chemistry Institute

**Published:** 2007-05

**Authors:** Erin E. Dooley

The Green Chemistry Institute (GCI) was formed in 1997 and became part of the American Chemical Society (ACS) in 2001. The institute works to advance the growth of green chemistry and green engineering, a movement to develop and implement manufacturing processes that are both economically sound and environmentally sustainable. Information on the institute’s different activities and a wealth of resources are available at **http://chemistry.org/greenchemistryinstitute**.

The GCI site includes an overview of green chemistry and green engineering, and outlines 12 principles underlying each. The homepage also has a News section, divided into Green Chemistry Updates and Headlines Around the World. The updates link to information on ACS events (such as roundtables, workshops, and meetings), announcements of new resources in the field, and other notable happenings. The Research section of the site has details about GCI funding opportunities and fellowships.

The Education section provides educational resources categorized by four levels: graduate, undergraduate, high school, and primary school. In the Undergraduate section, for example, are green engineering modules for chemical engineering courses that include problems and case studies that can be put into use in traditional classes. Also on offer is the ACS publication *Greener Approaches to Undergraduate Chemistry Experiments*, a collection of 14 laboratory activities that use green chemistry principles to investigate typical topics in undergraduate chemistry. The coursebook *Introduction to Green Chemistry* is also available in this section, as is a group of case studies based on the winners of the Presidential Green Chemistry Challenge Awards. A list of postsecondary schools in the United States and abroad that offer green chemistry programs is also featured in the Education section.

The Industrial Implementation section of the site offers a glimpse into resources the institute offers to companies. The GCI conducts professional training in green chemistry principles, applications, methods, tools, and techniques. It also provides technical assistance in the forms of opportunity assessments, databases of new technologies, and benchmarking. Finally, the institute furnishes companies ways to gain recognition for their green efforts through media and community outreach, the Presidential Green Chemistry Challenge Awards, and an annual Green Chemistry and Engineering Conference.

The International Cooperation section includes links to the 23 international chapters of the GCI. These chapters conduct educational programs in their countries, host green chemistry events, and produce publications. One of the three chapters in the United Kingdom has developed a green chemistry program targeted at consumers and retailers.

The Resources section of the GCI site indexes links under seven categories. Among other offerings in the Electronic Tools category is the Green Chemistry Resource Exchange, a searchable database of news articles, journal articles, reports, and presentations. By joining the exchange, visitors can add new entries to the database and receive updates on advances in the field.

## Figures and Tables

**Figure:**